# A case of primary vasoproliferative tumor with full-thickness macular hole

**DOI:** 10.3205/oc000189

**Published:** 2022-02-08

**Authors:** Pukhraj Rishi, Janani Sreenivasan

**Affiliations:** 1 Shri Bhagwan Mahavir Vitreoretinal Services, Sankara Nethralaya, Chennai, Tamil Nadu, India

**Keywords:** retinal vasoproliferative tumor, full-thickness macular hole, remote macular complication, intraocular tumors, macula, retina

## Abstract

A 53-year-old female presented with defective vision in the left eye of one week duration with a best-corrected visual acuity (BCVA) of 6/9. Fundus examination showed a primary retinal vasoproliferative tumor (RVPT) at the inferotemporal quadrant and a small full-thickness macular hole (MH). The patient underwent cryotherapy for RVPT, followed later by vitrectomy with internal limiting membrane peeling and gas tamponade. The tumor regressed and the MH was closed with BCVA of 6/9. RVPT can present with remote macular complications like epiretinal membrane, cystoid macular edema, macular exudates, and rarely full-thickness MH. Management should be directed towards the tumor as well as the complication.

## Introduction

Retinal vasoproliferative tumors (RVPT) are uncommon retinal lesions that may occur in isolation (primary) or in association with another ocular condition (secondary) [1], [2], [3], [4], [5]. They typically appear as globular or dome-shaped yellow-pink lesions, arising in the peripheral retina, most commonly the inferotemporal quadrant [1], [2], [3], [4], [5]. Apart from local complications like hemorrhages, yellowish sub-retinal exudates, and exudative retinal detachment (rare), they are known to cause vision drop due to remote macular complications like epiretinal membrane, cystoid macular edema, and macular exudates [1], [2], [3], [4], [5]. Published reports have mentioned that RVPT is an often overlooked cause of macular dysfunction, and clinicians should be vigilant in peripheral fundus examination for conditions that can affect the macula, such as RVPT [1]. In this case report, we describe a case of primary RVPT associated with full-thickness macular hole (MH) managed with cryotherapy of the tumor and MH surgery with good outcome.

## Case description

**Figure 1:**
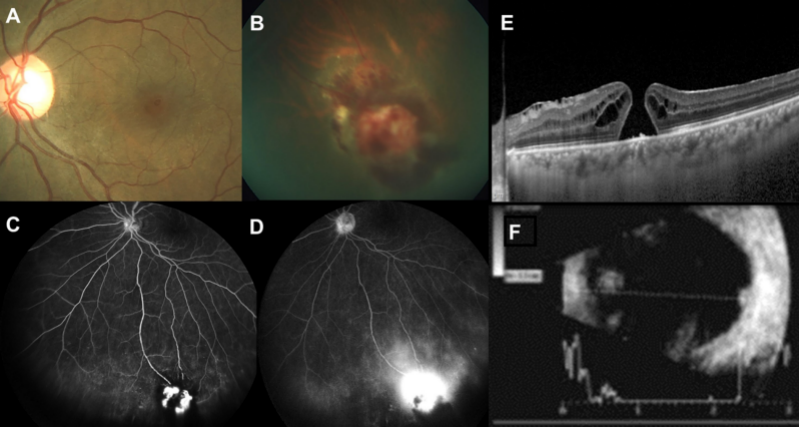
A) Color fundus photo of the left eye shows a small full-thickness macular hole. B) The inferotemporal quadrant shows a retinal vasoproliferative tumor, seen as a pinkish red mass with retinal hemorrhages. C, D) The fundus fluorescein angiography in the early phase showed hyperfluorescence corresponding to the tumor with a few areas of blocked fluorescence due to hemorrhages with progressive leakage at the late phase. E) Spectral domain optical coherence tomography of the left eye shows a small full-thickness macular hole with cystic spaces at the edges of the hole. F) Ultrasound revealed an acoustically solid tumor with medium to high internal reflectivity with tumor dimensions of 3.8 mm (longitudinal basal diameter) x 2.6 mm (vertical basal diameter) x 1.6 mm (apical thickness).

**Figure 2:**
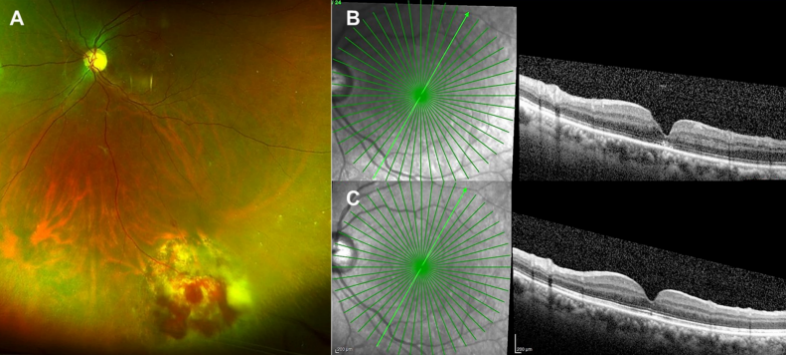
A) Pseudocolor fundus photograph of the left eye shows regressed retinal vasoproliferative tumor with chorio-retinal atrophic patches, secondary to cryotherapy. B) Spectral domain optical coherence tomography of the left eye at 6 weeks postoperative period shows closed macular hole (type 1 closure) with slightly altered foveal contour and few ellipsoid zone defects (EZ). C) At 6 months postoperative period, the macular hole is closed with restoration of the foveal contour and near normal EZ.

A 53-year-old, apparently healthy female presented with complaint of distortion in vision of the left eye of a week’s time. She was diagnosed elsewhere as MH in the left eye and referred for MH surgery. She underwent hysterectomy for menorrhagia two years ago elsewhere; systemic history was otherwise unremarkable. BCVA was 6/6 in the right eye and 6/9 in the left eye, N8. Right eye examination was essentially normal. The left eye had early lens changes with the intraocular pressure of 12 mm Hg. Fundus examination of the left eye (Figure 1A, B (Fig. 1)) showed a small full-thickness MH and a yellowish-pink, slightly elevated lesion in the inferotemporal quadrant with retinal hemorrhages. Ancillary investigations in the right eye were unremarkable. Ultrasound (Figure 1F (Fig. 1)) revealed an acoustically solid tumor with medium to high internal reflectivity. The tumor dimensions were 3.8 mm (longitudinal basal diameter), 2.6 mm (vertical basal diameter), and 1.6 mm (apical thickness). In the left eye, fundus fluorescein angiography (Figure 1C, D (Fig. 1)) in the early phase showed hyperfluorescence corresponding to the tumor with a few areas of blocked fluorescence due to hemorrhages with progressive leakage at the late phase. Spectral domain optical coherence tomography (SD-OCT) (Figure 1E (Fig. 1)) revealed a full-thickness MH with few cystic spaces at the edge of the hole. The tumor was managed with triple freeze-thaw transconjunctival cryotherapy. The patient was reviewed 2 weeks later, and the tumor was found to be regressing. Subsequently, the patient underwent 25G vitrectomy with internal limiting membrane peeling and perfluoro propane (C3F8) gas tamponade. She was advised to maintain prone positioning for 2 weeks. The intraoperative and early postoperative period were uneventful. At 6 weeks postoperative period, the tumor regressed completely (Figure 2A (Fig. 2)), the patient’s BCVA maintained at 6/9 with near vision improved to N6. SD-OCT showed closed MH with mildly altered foveal contour and few EZ (ellipsoid zone) defects (Figure 2B (Fig. 2)). At 6 months postoperative period, the patient was clinically stable with maintained BCVA, and SD-OCT showed completely restored foveal contour and EZ (Figure 2C (Fig. 2)).

## Discussion

RVPT are peripheral retinal vascular lesions that were described as a distinct clinical entity by Shields et al. in a series of 12 patients in 1983 [6]. However, based on recently published reports on histopathology and molecular findings, ‘reactive retinal astrocytic tumor’ has been proposed as an alternate terminology, as the predominant components of the tumors are astrocytic and glial cells, with a minimal vascular component [7].

RVPT were classified by Shields et al. as primary (idiopathic) and secondary when they occur secondary to pre-existing ocular conditions like retinitis pigmentosa, familial exudative vitreoretinopathy, Coats disease, intermediate uveitis, retinal vasculitis, previous retinal detachment surgery, etc. [1]. Idiopathic RVPT are usually unilateral, solitary, and localized, whereas secondary tumors are often diffuse, multiple, and rarely bilateral [1]. With respect to systemic association, Sickle cell disease and neurofibromatosis type 1 are important [5], [8].

Clinically, RVPT appears as a yellow-red, often ill-defined retinal mass with a mildly dilated feeding retinal artery and draining vein [1], [2], [3], [4], [5]. Ultrasound shows an acoustically solid tumor with medium to high internal reflectivity [1], [4], [5]. Widefield FFA can aid in the diagnosis, which reveals early filling in the arterial phase with increasing hyper fluorescence and late leakage [5]. Although RVPT is a benign tumor, it can produce profound visual loss related to remote effects of the tumor, including macular exudation, cystoid macular edema, vitreous hemorrhage, and epiretinal membrane [1], [4], [5]. In their study of 275 patients, Shields et al. noted macular exudates in 23%, cystoid macular edema in 32%, and epiretinal membrane in 20% of their patients [1]. Very rarely, RVPT can cause neovascular glaucoma and may lead to a painful blind eye [1], [5]. Optical coherence tomography is helpful to assess these macular pathologies. The clinical features and the imaging findings in this case are compatible with the published reports in the literature. MH has been reported to be associated with other intraocular tumors like posterior uveal melanoma and congenital simple hamartoma [9], [10]. This is the first case report from Asia reporting MH in association with primary RVPT, to the best of our knowledge. The likely cause of MH formation could be due to altered vitreo-retinal interface or de-roofing of the cystoid macular edema secondary to RVPT [9]. On the contrary, considering the age of the patient, idiopathic etiology for MH could not be ruled out.

The most important differential diagnosis is retinal capillary hemangioblastoma, which manifests with more prominent retinal vessel dilatation and tortuosity [1], [4], [5]. Other diseases in the list include amelanotic choroidal melanoma, peripheral exudative hemorrhagic choroidopathy (PEHCR), and inflammatory granuloma (tuberculosis, sarcoidosis) [4], [5]. Melanoma can be differentiated by its typical choroidal location and echographic features (high surface reflectivity, low to medium internal reflectivity with acoustic hollowing, choroidal excavation). PEHCR is characterized by a homogenous deep brown mass in the periphery with exudative and hemorrhagic changes, blockage on fundus fluorescein angiography, and echodensity with retraction cleft in ultrasound. Inflammatory granulomas exhibit vitreous cells, (can be seen in RVPT also), but look paler and lack the typical vascular component [5].

There are no clear-cut guidelines in the management of RVPT [5]. The presence of macular complications is considered as an indication for treatment according to a few authors [2]. The various management options include cryotherapy, laser photocoagulation, radiotherapy, photodynamic therapy, surgical excision for the tumors, anti-VEGF injections, and dexamethasone implant for macular edema [1], [2], [3], [4], [5]. The triple freeze-thaw transconjunctival cryotherapy under observation through binocular indirect ophthalmoscopy is the most popular treatment modality for RVPT with a thickness of less than 2 mm, favored by peripheral location [3], [5]. The treatment goal is to freeze the entire tumor, allowing slow thawing and repeating the whole process two or three times. More than one cryotherapy session can be necessary to achieve complete tumor involution, especially in thick tumors [5]. This can be combined with anti-VEGF or dexamethasone implant injection for macular edema/exudates [5]. Complications of cryotherapy include the persistence of macular edema and the occurrence of retinal detachment arising in a retinal tear adjacent to the scarred area [5]. For larger tumors (>2.5 mm thickness) and those associated with retinal detachment, plaque radiotherapy is preferred [4], [5]. In this patient, transconjunctival cryotherapy was done to the tumor first, followed by MH surgery to reduce the risk of intraoperative complications like vitreous hemorrhage, retinal breaks, and exudative retinal detachment.

## Conclusion

RVPT can cause vision loss due to remote macular complications despite their peripheral location. This case report underscores the importance of thorough peripheral retinal examination in cases of macular pathology to rule out conditions like RVPT, as well as the need to address both the tumor and the complication in the management plan.

## Notes

### Competing interests

The authors declare that they have no competing interests.
